# An unusual case report of basal cell adenoma: A Diagnostic Enchanter

**DOI:** 10.4317/jced.51642

**Published:** 2014-12-01

**Authors:** Swati Gupta, Shweta Rehani, Monica Mehendiratta, Madhumani Kumra, Ramakant Gupta, Kanu Jain

**Affiliations:** 1 MDS Postgraduate student. Department of Oral Pathology, Microbiology and Forensic Odontology; 2 Reader. Department of Oral Pathology, Microbiology and Forensic Odontology; 3 Professor and HOD. Department of Oral Pathology, Microbiology and Forensic Odontology; 4 Lecturer. Sudha Rustagi College of Dental Sciences and Research; 5 Senior lecturer. Department of Oral Pathology, Microbiology and Forensic Odontology

## Abstract

Oral lesions show a wide range of biologic behaviours. There are various lesions which may mimic others and present in such an unusual manner thus making them very difficult to diagnose clinico-pathologically. An accurate diagnosis is not only important for correct treatment planning but also for determination of prognosis. Thus, it is very important for a surgical pathologist to be aware of the various atypical presentations of the lesions. The present unusual case report of basal cell adenoma occurring on upper lip with frank areas of calcifications and abundant inspissated mucoid secretions is an example of one such case. BCA is an uncommon benign epithelial salivary gland neoplasm. It is one of the nine subcategories of salivary gland epithelial tumours according to WHO 2005 classification of salivary gland tumors. It is composed of basaloid cells organized with a prominent basal cell layer and distinct basement membrane-like structure and no myxochondroid stromal component as seen in pleomorphic adenomas. To our best knowledge, no case in English literature has been reported BCA with exuberant inspissated mucoid secretions and frank areas of calcifications to such a large extent and this is the first case to report the same.

** Key words:** Basal cell adenoma, calcifications, diagnosis, inspissated mucoid secretions, surgical pathologist.

## Introduction

Basal cell adenoma [BCA] is an uncommon benign epithelial salivary gland neoplasm [1-2%] of all salivary gland tumours (1), with majority arising in parotid glands of elderly patients (2). Histopathologically, it has characteristic uniform appearance which is dominated by basaloid cells (3). BCAs have various variants (3), including solid, trabecular, tubular and membranous type. Although BCAs are benign in nature, but few cases of membranous type of BCA have reported high recurrence rate [24%] (1). Malignant transformation of membranous BCA and hybrid tumours consisting of basal cell adenoma and adenoid cystic carcinoma has also been reported (2). Thus, making it more important to diagnose BCA and distinguishing it from other salivary gland neoplasms.

The categorization of any neoplasm whether benign or malignant, is crucial in terms of treatment planning, evaluation, and determination of prognosis. This relies upon the histopathological diagnosis which is made after the macroscopic and microscopic examination of the biopsy obtained. It is the responsibility of the surgical pathologists to diagnose as accurately as possible using all the appropriate diagnostic tools available.

Thus, intention of this case report is to report an atypical case of BCA on upper lip with frank areas of calcification and exuberant inspissated mucoid secretions which has never been reported by any author till now. At the first glance, these areas of calcifications and exuberant mucoid secretions were thought to be a tissue which has been burned due to technical errors. Later on, series of investigations and a careful examination led to the diagnosis of BCA. The aim of this paper is to make pathologists worldwide, aware of difficulties which may be encountered during the diagnosis of such an unusual case.

## Case Report

A 42 year old male reported to outpatient department in Sudha Rustagi College of Dental Sciences and Research, Faridabad, India; with a chief complain of a painful swelling on inner side of upper lip since six months. The history revealed an insidious, slow growing painless swelling since 3-4 years which became painful since 6 months. Pain was continuous, diffuse, dull and non- radiating in nature. There was no history of any associated trauma/ discharge/ any other swelling/ any dental treatment. The past medical/ dental/ personal history was non contributory. On extraoral examination, no evidence of swelling/ asymmetry or any other abnormality was detected. Intraoral examination demonstrated a well circumscribed, symmetric, dome-shaped sessile swelling was present on inner surface of upper labial mucosa. It was approximately 1.0 cm in diameter crossing midline, pale pink in colour with few red areas and smooth in surface texture. There was no blood/ pus discharge and surrounding mucosa was normal. On palpation, swelling was multinodular, firm in consistency, tender, non-fluctuant, non-compressible, non-mobile and had no localised raised temperature.

The swelling was provisionally diagnosed as pleomorphic adenoma and an excisional biopsy was performed under LA and was examined histopathologically.

**Figure 1:**
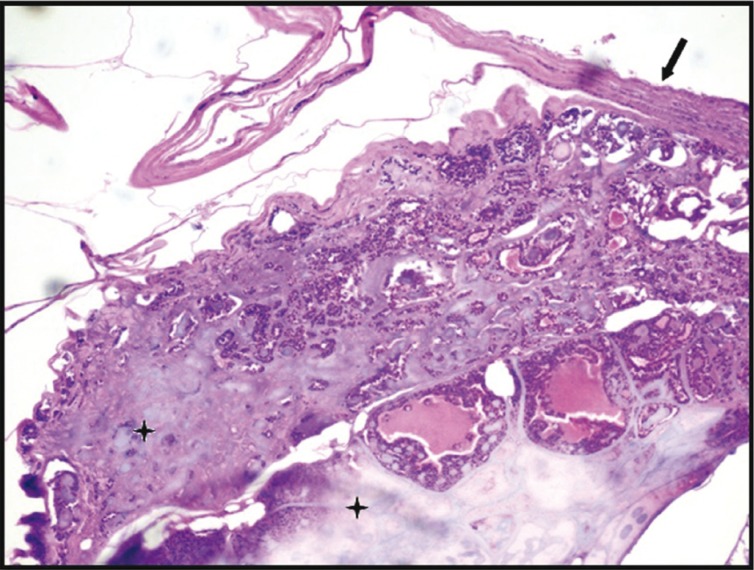
Photomicrograph showing encapsulated mass of tumour cells in varying patterns like glandular, ductal, nests and chords with minimal stroma and inspissated mucoid secretions (H&E staining, 10x). The arrow indicates capsule, star indicated inspissated mucoid secretions.

**Figure 2:**
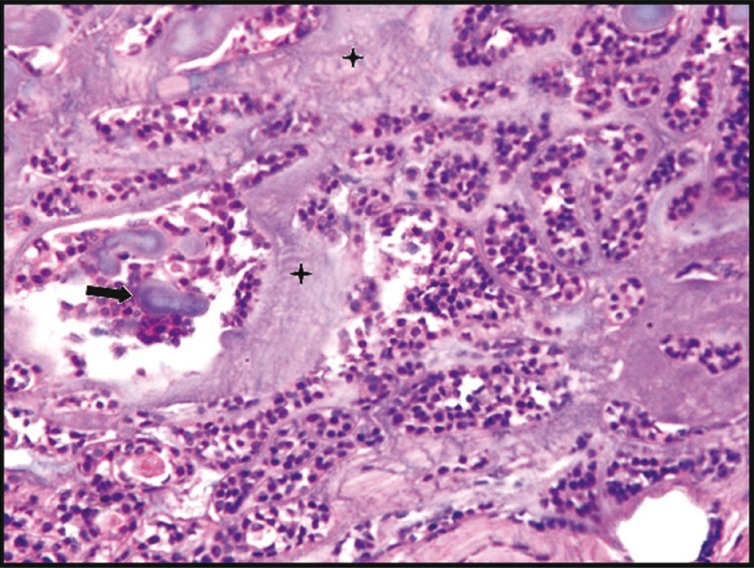
Photomicrograph showing mass of tumour cells arranged in varrying pattern with inspisated mucoid secretions and calcification foci (H&E staining, 40x). The arrow indicates calcification foci, star indicates inspisated mucoid secretions.

**Figure 3:**
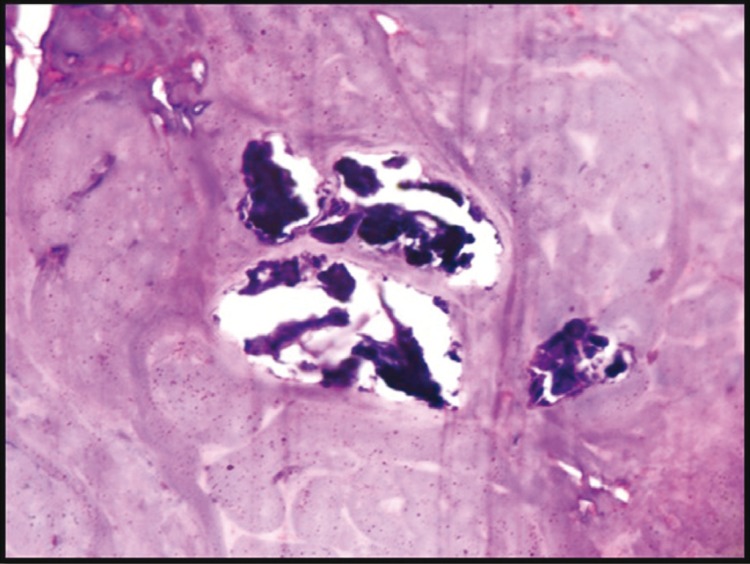
Photomicrograph showing calcification foci within inspisated mucoid secretions (H&E staining, 40x).

Macroscopically, the specimen was 1.0x 0.8x 0.7 cm in size, creamish red in color, firm in consistency and smooth in texture. Microscopically, H&E stained soft tissue sections showed well circumscribed and encapsulated tumour. In the centre of the tumour, exuberant, faintly basophilic mucoid secretions with numerous globular and highly basophilic areas of calcifications were seen. Mucoid secretions were surrounded by the isomorphic basaloid tumour cells, which were arranged in patterns of varying sizes and shapes such as glandular, ductal, nests and cords (Figs. 1-3). Basaloid cells exhibited hyperchromatic nuclei with scant eosinophillic cytoplasm. The PAS stained sections confirmed the inspissated mucoid secretions and presence of PAS positive eosinophillic hyaline layer that surrounded epithelial islands and separated these islands from one another.

Histopathologically the case was diagnosed as basal cell adenoma with atypical findings of presence of inspissated mucoid secretions and calcification foci.

## Discussion

The highlight of the present case report is the exuberant inspissated mucoid secretions and prominent calcification foci which were very misleading. For the first glance, the inspissated mucoid secretions resembled the burnt tissue which has been burnt during tissue processing due to negligence of laboratory technician. It was interpreted that probably during impregnation and embedding, the paraffin wax was kept at extremely high temperature, which could have caused the tissue to burn. The sections microscopically appeared to be degenerated with myxoid appearance and loss of cellular details as seen in a burnt tissue. Later, on interrogating the technician and enquiring about the processing technique used, it was found that no processing error was made. Further, it was revealed that the tissue posed a difficulty for the technician as well during microtomy as the tissue was firm. Since it was clear that tissue was not burnt, many serial sections were made and examined. Along with routine staining, PAS staining was also performed which confirmed the presence of inspissated mucoid secretions superimposed on the cellular structures.

Conventionally, inspissation is the process of thickening due to dehydration (4). In laboratory, this process is used while heating the media which consists of high protein content to isolate bacteria (4). Pathophysiologically in humans, inspissation of secretions may be commonly seen in cystic fibrosis and various diseases of respiratory tract or GIT (5). Pathologic inspissation occurs in the secretions which are long standing (5). It may also occur due to adsorption of the liquid components of these secretions by the surrounding structures (5). To our best knowledge, no case report in literature has been reported BCA with inspissated mucoid secretions to such a large extent. The present case is the first case to report the findings.

Antoniades D. *et al.* (6) presented a case of mucous retention cyst [MRC] with development of BCA on upper lip at 58th annual meeting and continuing education program of American academy of Oral and Maxillofacial Pathologists. MRC is a true cyst lined by uniform layer of cuboidal to low- columnar cells with occasional mucous cells within epithelium. Since BCAs are epithelial in origin (7) and the source of cells being intercalated and terminal ducts therefore, in the present case, it may be hypothesised that a long standing mucous retention cyst may have been transformed to BCA although patient was unable to recall the history of trauma.

Another obstacle encountered during the diagnosis of the present case was the presence of frank areas of calcifications. To our best knowledge, there are only three cases in English literature (8) which has reported microcalcifications in BCA, with none showing such frank areas of calcification. Various theories have been proposed till now to explain the mechanism of calcifications (9). These are based on two mechanisms: first, booster mechanism; second is seeding/ nucleating mechanism.

It may be hypothesized that inspissated mucoid secretions present in long standing mucocele, may have under-gone dystrophic calcifications leading to the frank areas of calcification. The mucoid secretions contains water [95%], most of which is bound in a viscoelastic gel containing mucins (10). The gel-forming mucins are high-molecular-weight glycoproteins (10) and may contain carbohydrates [such as L- fructose, mannose, galactose, galactosamine], sialic acids [N- acetylmuramic acid], unsulphated hyaluronic acid, or sulphate groups [such as chondroitin sulphate] (8,10). Although, chondroitin sulphate initially was thought to be a seeding agent, but due to its acidic nature, now it is considered as an inhibitor (8). The role of other mentioned components in calcification process, as seeding agents has already been known.

Since no such case with presence of such exuberant inspissated mucoid secretions and calcification foci in BCA have been reported in literature, our case having both is the first case reporting the same, and is thought to be extremely rare.

## References

[1] Jang M, Park D, Lee S R, Hahm C K, Kim Y, Kim Y (2004). Basal Cell Adenoma in the Parotid Gland: CT and MR Findings. Am J Neuroradiol.

[2] Crumpler C, Schafrenbrg J C, Reed RJ (1976). Monomorphic adenomas of salivary glands.
Trabecular-tubular, canalicular, and basaloid variants. Cancer.

[3] Nakabayashi M, Shomori K, Kiya S, Shiomi T, Nosaka K, Ito H (2010). Tubular-Trabecular Type Basal Cell Adenoma of the Parotid Gland: A Patient Report. Yonago Acta Medica.

[4] Perlman M, Williams J, Hirsch M, Bar-Ziv J (1975). Familial non cystic fibrosis mucous inspissation of respiratory tract. Arch Dis Child.

[5] Tucker JA, Spock A, Spicer SS, Shelburne JD, Bradford W (2003). Inspissation of pancreatic zymogen material in cystic fibrosis. Ultrastruct Pathol.

[6] Antoniades D, Markopoulos A, Zaraboukas T, Epivatianos A (2004). Mucus retention cyst with development of basal cell adenoma: report of a case. Oral Surg Oral Med Oral Pathol Oral Radiol Endodontol.

[7] Dardick I, Kahn HJ, Van Nostrand AW, Baumal R (1984). Salivary gland monomorphic adenoma. Ultrastructural, immunoperoxidase, and histogenetic aspects. Am J Pathol.

[8] Nakamura C, Kawakami T, Hasegawa H, Shigeo EDA (1987). Light and Electron Microscopic Studies of Microcalcifications Appearing in Monomorphic of Microcalcifications Adenomas. Matsumoto Shigaku.

[9] Irving J (1973). Theories of Mineralization of Bone. Clin Orthop Relat Res.

[10] Sonesson M (2011). On minor salivary gland secretion in children, adolescents and adults. Swed Dent J Suppl.

